# The Jichi Medical School (JMS) Cohort Study: Design, Baseline Data and Standardized Mortality Ratios.

**DOI:** 10.2188/jea.12.408

**Published:** 2007-11-30

**Authors:** Shizukiyo Ishikawa, Tadao Gotoh, Naoki Nago, Kazunori Kayaba, the Jichi Medical School (JMS) Cohort Study Group

**Affiliations:** 1 Department of Community and Family Medicine, Jichi Medical School.; 2 Wara National Health Insurance Hospital,; 3 Tsukude National Health Insurance Clinic,; 4 Joetsu Medical Center.

**Keywords:** cohort studies, multi-center studies, population-based studies, cardiovascular diseases, Japan

## Abstract

We have been conducting a cohort study titled the Jichi Medical School Cohort Study (The JMS Cohort Study) since 1992, which is aiming to clarify the risk factors of cardiovascular and cerebrovascular diseases in the Japanese. The baseline data were gathered from April 1992 through July 1995 in 12 rural districts using a legal mass screening system. The total samples were 12,490 males and females, and the overall response rate for the total population was 63%. The mean ages were 55.2 years for males and 55.3 years for females, respectively. Smoking rates were 50.5% and 5.5%, and drinking rates were 75.1% and 25.0% for males and females, respectively. We also examined the Standardized mortality ratios (SMRs) of the cohort subjects for 7.6 year follow-up period. The SMRs were 0.68 [95% confidence interval (CI): 0.59-0.78] for males and 0.73 (95% CI: 0.62-0.85) for females for the cohort subjects, whereas the SMRs were 1.00 (95% CI 0.97-1.04) for males and 1.06 (95% CI: 1.02-1.10) for females for all residents. In this article, we outlined the cohort study and showed general characteristics of the baseline data, and the SMRs of the subjects. We have been following the eligible subjects, and are preparing to show some prospective data regarding cardiovascular and cerebrovascular risks in the near future.

Cardiovascular diseases and cerebrovascular diseases (strokes) were the second and the third leading causes of mortality recently in Japan. Crude and age-adjusted mortality rates from stroke have declined rapidly during the recent two decades,^1^^,^^2^ but it still remains a major health problem. The main reason for the decrease in stroke mortality was due to the decrease of intracranial hemorrhages.^3^ Several Japanese studies have investigated the trends of stroke and cardiovascular diseases,^3^^,^^4^ and the relationship among these diseases and certain risk factors. Life style has been changing since the end of World War II, especially in the last few decades. Westernization of the diet, and decreasing hard labor due to automization are major changes.

It remains unclear why cardiovascular diseases mortality rates were lower, and stroke rates were higher in Japan than in Western countries. There have been many population-based cohort studies in Western countries,^5^^-^^8^ and several population based cohort studies have been conducted in Japan as well. Some of them were large-scale cohort studies, but the main outcomes were mostly mortality.^9^^-^^11^ In only a few population based cohort studies, the main outcome were incidence of strokes and cardiovascular diseases in Japan,^3^^,^^4^^,^^12^ which is to clarify the characteristics of strokes and cardiovascular diseases. Some of these studies in Japan, however, were conducted decades ago, and a study was required to reflect the current situation in Japan.

It is important that the study subjects represent general population when incidence rate is calculated and risks are estimated. The standardized mortality ratio (SMR) is helpful to make a guess to what extent the subjects represent general population. Some studies have examined SMR in Japan, but most of them reported the SMRs of workers migrants or specific cause mortality, such as stomach cancer. We could find only few studies about the SMR of population-based study, and one follow-up study were examined using the system of the annual health check-up mass screening examination. The study showed the SMR was about 0.4 in a city where response rate for the check-up was about 30%.^13^

In this article, we showed the general characteristics of baseline data, and also calculated the SMRs of all cause mortality of the participants of the JMS Cohort Study.

## MATERIALS AND METHODS

### Subjects

The JMS Cohort Study is a study that began in 1992 to clarify the relationship among life-styles, socio-economic factors, serum lipids, and other risk factors with strokes and cardiovascular diseases in 12 rural districts in Japan (Figure). Baseline data were collected between April 1992 and July 1995. Mass screening examinations for cardiovascular diseases have been conducted since 1983 in accordance with the health and medical service law for the aged, and we used this system to collect the data. In each community, a local government office sent personal invitations to all the subjects by mail. The subjects for the mass screening examinations were residents aged 40-69 years in Iwaizumi, Take, Kuze, Sakuma, Sakugi, Okawa, Ainoshima, and Akaike, and were aged 35 years and older in Wara. Subjects for other age groups were included in Yamato, Takasu, and Hokudan. As a result, 12,490 subjects were eligible (4,911 males and 7,579 females) for all ages (19-93 years of age), 1,119 for Iwaizumi, 2,851 for Tako, 2,404 for Yamato, 450 for Kuze, 1,478 for Takasu, 1,371 for Wara, 306 for Sakuma, 1,129 for Hokudan, 394 for Sakugi, 214 for Okawa, 136 for Ainoshima and 638 for Akaike. There were 10,612 (4,099 males and 6,513 females) aged 40-69 years.

**Figure.**
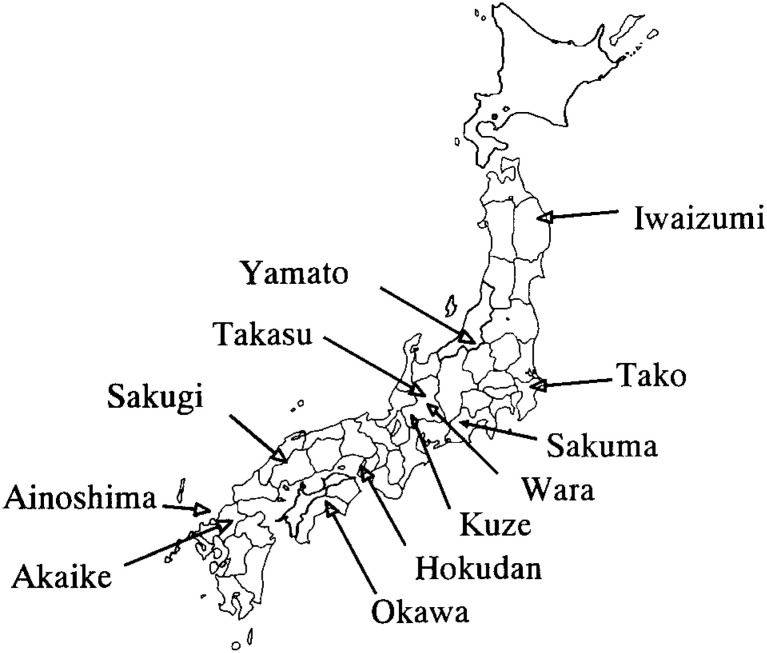
The twelve districts participating in the JMS Cohort Study

### Baseline data collection

Some basic data were obtained in all districts, and some other data were optionally used in each district. Body height and body weight, blood pressure, electrocardiogram, urine protein, sugar, and occult blood urinalysis, erythrocyte count, hemoglobin, hematocrit, total cholesterol, high density lipoprotein (HDL) cholesterol from a blood sample, habitual food consumption, smoking status, drinking status, past medical history, family history, occupation, physical activity, and job stress data were obtained from questionnaires.

Data were obtained for three years in Iwaizumi, Tako, Yamato, Takasu, Wara, Sakuma, Sakugi, Akaike, and for a year in Kuze, Hokudan , Okawa, and Ainoshima from 1992 to 1995, and if several data were obtained for an identical subject during the period, the first one was used as a base-line data. Life style was not considered to change over three years of collecting baseline data. To obtain information using a uniform method, we established a central committee, that is composed of the chief medical officers from all the participating districts, which developed a detailed manual for data collection. Body height was measured in stocking feet. Body weight was recorded with the subjects clothed, and 0.5 kg in summer and 1.0 kg in the other seasons was subtracted from the recorded weight. Body mass index were calculated as weight (kg) / height (m)^2^. The systolic and diastolic blood pressures were measured with a fully automated sphyngomanometer, BP203RV-II (Nippon Colin, Komaki, Japan), placed on the right arm of a seated subject who had rested in the sitting position for 5 minutes before the measurement. Electrocardiograms were measured with machines used in the mass screening program in each district. Protein, glucose and occult blood from urine were determined by urinalysis with a dip stick.

Blood samples were obtained in the morning after an overnight fast, except in Tako, Hokudan and Ainoshima, where the samples were taken without consideration of the most recent food intake, and the interval time between the last food intake and blood collection was recorded. Blood samples were drawn from the antecubital vein of a seated subject with minimal tourniquet use. Specimens were collected in siliconized vacuum glass tubes containing 1/10 volume of 3.8 % trisodium citrate (fibrinogen and factor VII), sodium fluoride (blood glucose), or no additives (lipids). Tubes were centrifuged at 3,000 g for 15 minutes at room temperature. After separation, the serum samples were stored at 4°C in refrigerated containers if analyses were to be performed within a few days. Otherwise, the samples were frozen until analysis. Plasma samples were stored in refrigerated containers with dry ice for a maximum of 6 hours, and then frozen as rapidly as possible to -80°C for storage until laboratory tests were performed.

Total cholesterol and triglyceride were measured by an enzymatic method (Wako, Osaka, Japan; inter assay coefficient of variation (CV): 1.5 % for total cholesterol and 1.7 % for triglyceride). HDL-cholesterol was measured using the phosphotungstate precipitation method (Wako, Osaka, Japan; interassay CV: 1.9 %). Blood glucose was measured via an enzymatic method (Kanto Chemistry, Tokyo, Japan; interassay CV: 1.9 %.

Lipoprotein(a) (Lp(a)) levels were measured using an enzyme-linked immunosorbent assay (ELIZA) kit (Biopool, Uppsala, Sweden; interassay CV: 3.51 %). The minimum detectable Lp(a) level was 1 mg/dl and undetectable Lp(a) values were recorded as 0.5 mg/dl. Fibrinogen levels were determinated with a one-stage clotting assay kit (Data-Fi, Dade, Miami, Florida, U.S.A.; interassay CV: 2.5 %). Factor VII activity was measured with a chromogenic assay using a human placenta-derived calcified thromboplastine reagent (Chromoquick, Behringwerke, Marburg, Germany), human factor VII-deficient plasma (Behringwerke, Barburg, Germany), and a chromogenic assay autoanalyzer (Behringwerke, Marburg, Germany; interassay CV: 3.8 %).

Blood variables except FVII activity, red cell count, hemoglobin and hematcrit, were sent to and measured at the central SRL laboratory (Tokyo, Japan), a commercial hematology laboratory which was standardized by the Lipid Standardization Program, Center for Disease Control and Prevention, Atlanta, Georgia. Red cell count, hemoglobin and hematocrit were measured, and urinalyses with a dip stick were recorded in each laboratory in the districts.

Information about medical history and life style was obtained with a questionnaire. Habitual food intake was assessed using a five-level scale intake frequency, or preference for salty or greasy food. Questions about past or present illness were asked and if present, whether the subject took medicine or not was confirmed. Smoking status was judged as smoking, ex-smoking or never, and if smoking or ex-smoking, the number of cigarettes per day was recorded. Drinking status was classified as drinking, ex-drinking or never, and if drinking or ex-drinking, the frequency and quantity of drinking alcohol consumed were noted. Educational attainment was indexed by the total number of years in school. Menopausal status was asked of all women, whether menopausal state or not, and if menopausal, whether it was a natural or surgical menopause. As for employment status, the type of job and management status were recorded. Job characteristics were examined through questions developed from the MOPSY of WHO’s MONICA project^14^ via the questionnaire, which was based on a demand-control model involving two primary factors of job-related stress: job demands and job control (i.e., decision latitude). Physical activity was estimated by calculating the weighted sum of hours spent at five levels; 1.0 for sedation including sleeping, 1.1 for quiet working such as working in a sitting position, 1.5 for a light level of working such as working in a standing position, 2.4 for a moderate level of working, and 5.0 for heavy work, during a normal working day using the Framingham Study questionnaire.^15^

### Informed consent

Written informed consent for the study was obtained individually for the responders of the mass screening examination health check-up. We explained that we would gather data using the questionnaire and blood samples, would be following-up health status, and check the medical records in hospitals the subject visited if stroke or myocardial infarction were suspected to have occurred. All the responders agreed to join the study.

### The follow-up system

We used the mass screening examination system to obtain the baseline data for the cohort study, and were mainly using this system to try and check on all the subjects every year. We asked the subjects directly whether they have the history of stroke and cardiovascular diseases after enrolling, and if they had, asked which hospital and when they visited to ascertain the incidence of the diseases. For subjects who did not have the screening examination, we contacted them by mail or phone. We also checked the medical records if the subjects went to any hospitals. Public health nurses also visited the subjects to obtain information.

If an incident case was suspected, we filled out a form and duplicated computer tomography films for stroke, or electrocardiograms for myocardial infarction. Death certificates were collected at the public health centers with permission of the Agency of General Affairs and the Ministry of Health Labor and Welfare. Data on emigrants for the study subjects were obtained by each municipal government annually.

### Diagnostic criteria

The diagnosis was determined independently by the diagnosis committee, which is composed of radiologists, neurologists and cardiologists. Diagnosis of stroke was determined by the criteria of the Yanagawa group for stroke in the Ministry of Health and Welfare; existing a focal and unconvulsive neurological deficit lasting for 24 hours and longer with a clear onset of disease.^16^ Diagnosis of myocardial infarction was determined by the criteria of WHO’s MONICA project.^17^

### Statistical analysis

The unpaired t-test was used for comparing the mean values of variables, and regression analysis coefficiency was calculated for cross-sectional analysis. Analysis of variance was used for calculating the variance in categorical data. The SMR with 95% confidence interval (CI) for the subject participants was calculated for all deaths during follow-up periods in each community using age-specific mortality rate for the national population in 1995 as a standard. Similarly, SMR for all the residents aged 40 years and over was calculated. Overall SMRs for the subject participants and for all the residents were also calculated.^18^ These analyses were done using SAS software version 8.2.

## RESULTS

### General characteristics

The number of the cohort subjects and response rate for the check-up for each community for the age 40-69 years are shown in Table 1. The response rate for the check-up for all communities was 63%. The response rates for each community were: 77% for Iwaizumi, 65% for Tako, 78% for Yamato, 69% for Takasu, 90% for Wara, 79% for Kuze, 88% for Sakuma, 30% for Hokudan, 40% for Sakugi, 66% for Okawa, 26% for Akaike, and 44% for Ainoshima.

**Table 1.** The cohort subjects and response rates in the JMS Cohort Study

Region	Male (n)	Female (n)	Total (n)	Response rate*
Iwaizumi	398	720	1,118	77%
Tako	1,149	1,702	2,851	65%
Yamato	679	1,554	2,233	78%
Kuze	166	284	450	79%
Takasu	629	797	1,426	69%
Wara	615	756	1,371	90%
Sakuma	107	199	306	88%
Hokudan	511	578	1,089	30%
Sakugi	165	229	394	40%
Okawa	95	119	214	66%
Ainoshima	71	65	136	44%
Akaike	223	415	638	26%

Total	4,911	7,579	12,490	63%

* Response rate were calculated for the subjects aged 40-69 years in all communities.

Subjects aged 70 years and over were included in Takasu, Wara and Hokudan.

The total number of the JMS Cohort Study subjects was 12,490 (4,911 males and 7,579 females) in the 12 districts. The mean ages were 55.2 years for males and 55.3 years for females. The mean BMIs were 23.0 kg/m^2^ for males and 23.2 kg/m^2^ for females. Systolic and diastolic blood pressures were 131.4 mmHg and 79.2 mmHg for males, and 128.3 mmHg and 76.3 mmHg for females, respectively. Total cholesterol and HDL-cholesterol levels were lower in males (total cholesterol: 184.9 mg/dl, HDL-cholesterol: 48.8 mg/dl) than in females (196.7 mg/dl, 52.6 mg/dl). Triglyceride levels were higher in males (127.8 mg/dl) than in females (109.6 mg/dl). Blood glucose levels were higher in males (105.9 mg/dl) than in females (100.9 mg/dl). As for smoking, the proportions of current smokers, ex-smokers and nonsmokers were 50.5%, 28.3% and 21.2% for males and 5.5%, 2.8% and 91.7% for females, respectively. The proportions of current drinkers, ex-drinkers and nondrinkers were 75.1%, 3.7% and 21.2% for males and 25.0%, 1.5% and 73.5% for females, respectively (Table 2).

**Table 2.** General characteristics of the JMS Cohort Study from 1992-1995 in Japan.

	Male			Female		
	
	n	Mean	SD	n	Mean	SD
Age (years)	4,911	55.2	12.0	7,579	55.3	11.4
Systolic blood pressure (mmHg)	4,706	131.4	20.5	7,342	128.3	21.1
Diastolic blood pressure (mmHg)	4,706	79.2	12.3	7,342	76.3	12.1
Total cholecterol (mg/dl)	4,839	184.9	34.1	7,495	196.7	34.8
HDL-cholesterol (mg/dl)	4,840	48.8	13.3	7,495	52.6	12.5
Triglicerides (mg/dl)	4,839	127.8	86.7	7,494	109.6	67.6
Blood sugar (mg/dl)	4,840	105.9	31.2	7,476	100.9	22.6
BMI (kg/m^2^)	4,689	23.0	2.9	7,297	23.2	3.2

Smoking status						
Current smoker (%)	2,306	50.5		386	5.5	
Ex-smoker (%)	1,292	28.3		196	2.8	
Non-smoker (%)	967	21.2		6,418	91.7	
Total (%)	4,565	100.0		7,000	100.0	
Alcohol drinking status						
Current drinker (%)	3,338	75.1		1,712	25.0	
Ex-drinker (%)	162	3.7		103	1.5	
Non-drinker (%)	943	21.2		5,023	73.5	
Total (%)	4,443	100.0		6,838	100.0	

SD: standard deviation

The mean and standard deviation (SD) of the variables in each district for males and females are shown in Tables 3 and 4. Systolic blood pressure levels were higher in Ainoshima and Iwaizumi, and lower in Yamato and Wara for males. Diastolic blood pressure levels were higher in Ainoshima, Akaike, Iwaizumi and Sakugi, and lower in Yamato and Wara for males. Total cholesterol levels were lower, and BMI levels higher, in Iwaizumi than in other districts for males. Systolic and diastolic blood pressure levels were higher in Iwaizumi, Takasu and Sakugi, and lower in Yamato. Total cholesterol levels were lower in Iwaizumi.

**Table 3.** Means and standard deviations of the total subjects in each district of the JMS Cohort Study in males from 1992-1995.

Male	Age (years)			SBP (mmHg)			DBP (mmHg)			Total cholesterol (mg/dl)			HDL-cholesterol (mg/dl)			Triglycerides (mg/dl)			Blood sugar (mg/dl)			BMI (kg/m^2^)		
							
	n	Mean	SD	n	Mean	SD	n	Mean	SD	n	Mean	SD	n	Mean	SD	n	Mean	SD	n	Mean	SD	n	Mean	SD
Iwaizumi	398	57.9	7.9	396	138.8	21.8	396	82.6	12.6	398	179.2	32.1	398	51.8	14.4	398	129.3	78.3	398	118.7	42.9	396	24.2	2.9
Tako	1149	55.5	8.7	1111	132.5	17.3	1111	79.6	10.6	1139	185.6	34.4	1139	47.5	12.2	1139	147.0	93.9	1139	115.1	35.4	1110	23.1	2.8
Yamato	745	50.5	14.1	745	123.9	17.9	745	76.8	12.0	741	177.9	31.6	741	50.3	13.2	741	129.7	78.1	744	113.3	34.2	745	22.7	2.8
Kuze	166	58.0	8.8	165	131.8	20.1	165	79.1	12.1	166	181.8	34.7	166	49.0	13.4	166	135.5	108.9	163	98.5	35.9	165	22.8	2.8
Takasu	637	53.9	14.2	594	132.9	21.1	594	80.3	11.9	603	184.1	34.7	603	48.6	13.7	603	116.0	93.9	603	97.9	20.4	582	23.2	2.9
Wara	615	58.3	13.1	510	126.7	20.2	510	75.7	12.0	615	187.5	32.9	615	47.3	13.3	615	119.5	88.3	615	96.6	19.6	510	22.3	2.7
Sakuma	107	64.7	9.1	107	128.1	24.6	107	76.1	12.9	107	179.9	33.9	107	49.2	12.6	107	121.0	79.3	107	94.1	12.5	107	21.9	2.7
Hokudan	540	53.2	14.2	540	133.2	21.8	540	78.9	13.4	537	187.6	34.2	537	47.1	12.3	537	113.8	67.5	537	94.6	19.2	538	22.9	3.0
Sakugi	165	56.1	9.1	156	136.1	21.0	156	82.0	13.0	158	202.4	32.3	158	56.1	16.8	157	107.5	68.5	158	100.1	19.2	153	23.2	2.8
Okawa	95	58.1	8.8	91	130.1	25.5	91	78.8	14.6	87	178.1	31.0	87	49.0	13.6	87	106.1	82.0	87	103.4	47.0	91	23.5	4.0
Ainosima	71	58.5	7.6	70	141.5	23.1	70	83.0	14.5	71	183.0	34.6	71	44.7	10.5	71	125.5	85.7	71	105.2	17.1	70	22.4	3.1
Akaike	223	54.8	10.0	221	136.1	21.8	221	83.1	12.3	218	198.3	38.0	218	48.9	14.1	218	110.5	85.4	218	102.7	23.0	222	23.0	2.8
Total (male)	4911	55.2	12.0	4706	131.4	20.5	4706	79.2	12.3	4840	184.9	34.1	4840	48.8	13.3	4839	127.8	86.7	4840	105.9	31.2	4689	23.0	2.9

SBP: systolic blood pressure, DBP: diastolic blood pressure, BMI: body mass index

**Table 4.** Means and standard deviations of the total subjects in each district of the JMS Cohort Study in females from 1992-1995.

Female	Age (years)			SBP (mmHg)			DBP (mmHg)			Total cholesterol (mg/dl)			HDL-cholesterol (mg/dl)			Triglycerides (mg/dl)			Blood sugar (mg/dl)			BMI (kg/m^2^)		
							
	n	Mean	SD	n	Mean	SD	n	Mean	SD	n	Mean	SD	n	Mean	SD	n	Mean	SD	n	Mean	SD	n	Mean	SD
Iwaizumi	721	56.9	8.0	719	133.7	22.3	719	79.6	12.2	719	193.4	31.5	719	54.0	12.3	719	121.0	71.6	719	110.5	29.5	719	25.0	3.7
Tako	1702	55.6	8.6	1642	128.9	17.0	1642	76.2	10.1	1690	197.1	33.8	1690	51.8	12.0	1690	125.5	72.3	1689	109.8	25.2	1643	23.1	2.8
Yamato	1659	52.4	12.8	1659	119.5	19.4	1659	73.6	12.4	1657	191.4	35.5	1657	53.4	12.6	1656	109.3	73.5	1658	101.9	22.7	1659	23.0	3.3
Kuze	284	57.2	8.4	283	129.7	19.9	283	76.1	11.7	284	202.5	34.8	284	51.2	12.1	284	122.4	84.7	273	93.3	14.4	281	23.5	3.4
Takasu	841	53.4	14.5	790	133.3	23.3	790	78.6	13.3	808	190.7	35.2	808	51.9	12.1	808	90.5	53.5	801	92.6	15.3	747	23.2	3.2
Wara	756	58.3	13.0	659	129.0	22.1	659	75.7	12.7	756	195.3	32.4	756	50.1	11.7	756	100.0	55.3	756	92.9	16.3	659	22.5	3.0
Sakuma	199	62.1	9.5	199	131.3	21.0	199	76.6	12.1	198	205.3	32.7	198	52.9	12.8	198	109.6	57.9	198	95.9	18.5	199	22.2	2.9
Hokudan	589	54.9	12.3	587	131.9	21.7	587	76.5	12.3	586	205.9	36.0	586	52.8	12.3	586	101.1	60.1	585	92.3	13.8	586	23.0	3.1
Sakugi	229	57.8	8.8	213	134.3	21.3	213	78.6	12.0	217	209.3	33.9	217	59.4	14.4	217	92.7	50.9	217	97.7	22.4	212	22.8	2.6
Okawa	119	59.2	7.7	118	128.0	24.3	118	75.3	12.4	112	200.5	37.4	112	50.8	13.1	112	96.7	49.5	112	97.4	24.9	118	23.3	3.3
Ainosima	65	59.9	6.3	65	129.1	22.7	65	76.3	11.2	65	200.9	35.7	65	45.8	10.2	65	124.6	82.9	65	105.1	18.1	65	22.5	2.8
Akaike	415	54.7	9.3	408	129.9	21.6	408	77.9	12.1	403	207.6	35.6	403	55.7	14.2	403	87.0	51.5	403	95.6	13.2	409	22.6	3.0
Total (female)	7579	55.3	11.4	7342	128.3	21.1	7342	76.3	12.1	7495	196.7	34.8	7495	52.6	12.5	7494	109.6	67.6	7476	100.9	22.6	7297	23.2	3.2

SBP: systolic blood pressure, DBP: diastolic blood pressure, BMI: body mass index

### Standardized mortality ratio

The mean period of follow-up was 7.6 years for the cohort subjects, and 304 male and 217 female deaths were confirmed by death certificates. Residents aged 40 years and over comprised 56,815 (26,675 males and 30,140 females) in the participating communities, and the mean period of follow-up was 8.7 years. During the follow-up period, 4,001 male and 3,503 female deaths were confirmed by death certificates. The SMRs for male and female cohort subjects were 0.68 (95%CI: 0.59-0.78) and 0.73 (95%CI: 0.62-0.85), respectively. The SMRs for all the male and female residents were 1.00 (95%CI: 0.97-1.04) and 1.06 (95%CI: 1.02-1.10), respectively (Table 5).

**Table 5.** Standardized mortality ratios and their 95% confidence intervals for all residents and the cohort subjects.

Region	The Cohort subjects				All residents			
	
	Male		Female		Male		Female	
			
	SMR	(95% CI)	SMR	(95% CI)	SMR	(95% CI)	SMR	(95% CI)
Iwaizumi	0.93	(0.57 - 1.43)	1.10	(0.61 - 1.81)	1.28	(1.18 - 1.38)	1.29	(1.18 - 1.40)
Tako	0.59	(0.40 - 0.84)	0.69	(0.45 - 1.01)	1.05	(0.97 - 1.14)	1.20	(1.10 - 1.30)
Yamato	1.07	(0.70 - 1.55)	0.63	(0.37 - 1.00)	1.06	(0.97 - 1.17)	1.11	(1.01 - 1.23)
Kuze	0.59	(0.19 - 1.38)	0.67	(0.02 - 3.71)	1.18	(0.94 - 1.47)	1.00	(0.77 - 1.27)
Takasu	0.59	(0.39 - 0.86)	0.76	(0.48 - 1.14)	0.88	(0.72 - 1.08)	1.00	(0.80 - 1.24)
Wara	0.63	(0.47 - 0.83)	0.79	(0.54 - 1.13)	0.80	(0.66 - 0.98)	1.18	(0.97 - 1.43)
Sakuma	0.88	(0.40 - 1.67)	0.44	(0.09 - 1.28)	0.94	(0.70 - 1.24)	0.83	(0.57 - 1.16)
Hokudan	0.58	(0.39 - 0.85)	0.66	(0.39 - 1.06)	1.05	(0.96 - 1.15)	1.08	(0.98 - 1.18)
Sakugi	0.50	(0.14 - 1.28)	0.74	(0.27 - 1.61)	0.87	(0.72 - 1.05)	1.09	(0.89 - 1.34)
Okawa	0.36	(0.12 - 0.84)	0.42	(0.05 - 1.52)	0.66	(0.44 - 0.96)	0.80	(0.51 - 1.20)
Ainoshima	1.19	(0.39 - 2.78)	0.00		0.93	(0.60 - 1.39)	0.82	(0.50 - 1.27)
Akaike	0.38	(0.10 - 0.98)	0.57	(0.21 - 1.24)	1.19	(1.07 - 1.32)	1.04	(0.93 - 1.17)

Total	0.68	(0.59 - 0.78)	0.73	(0.62 - 0.85)	1.00	(0.97 - 1.04)	1.06	(1.02 - 1.10)

SMR: Standardized mortality ratio

CI: confidence interval

## DISCUSSION

The current study is a large-scale multi-center population-based cohort study to detect incidences of stroke and myocardial infarction. This study was comprised of 12 districts and the response rates varied among districts from 26% to 90%. However, the 12 districts were widely located from the northern area (Iwaizumi in Tohoku area) to the southern area (Akaike and Ainoshima in Kyushu) of Japan. The overall response rate for the study was 63%, which may contain some selection bias, and this figure was quite high. We showed outline of the whole study in this article, although we reported some cross-sectional data previously.^19^^-^^21^

As well as being a multi-center study, the current study has two other advantages. First, physicians in primary care did study and got information about disease incidences. It is very difficult in Japan to detect the all cases of stroke and myocardial infarction in a community-based cohort study, because community-based disease registration systems do not work. In addition, the medical insurance system allows patients go to a suitable hospital without an ambulance. Moreover, in clinics or small hospitals, medical records or clinical information are often inadequate to reach the diagnostic criteria for a study. We followed the subjects annually, and after obtaining annual information about hospitalization individually, physicians made diagnosis using medical records. Information about incidences from death certificates and surviving relatives was also available.

The second advantage is that, we collected information about some new risk factors in addition to classic ones. We got data not only about life-style such as food consumption, smoking and drinking status, but about social support, job stress, and for women, menopausal status. We measured not only serum lipids and blood sugar, but Lp(a), CRP, serum insulin, haemostatic factors such as fibrinogen, and factor VII. Some of these factors came to be considered risk factors of cardiovascular diseases recently, and no large-scale longitudinal study has been reported in Japan.

In Japan there were some cohort studies. In few decades ago, Hirayama et al. examined mortality with a large cohort of 265,000 subjects of which main outcome is mortality,^9^ Kodama et al. examined incidence of coronary heart diseases with a cohort of about 20,000 adult health subjects out of atomic bomb survivors,^4^ and The Hisayama Study examined incidence of stroke and cardiovascular diseases with a cohort in one town started in 1961 which ascertained diagnosis with clinical information and a high-rate of autopsy.^3^ Some other large-scale cohort studies started around 1990; the Japan Public Health Center-based Prospective Study on Cancer and Cardiovascular Diseases (JPHC) study which was public health center based study supported by Ministry of Health, Labor and Welfare examined risk for stroke, cardiovascular diseases and mortality;^11^ the Japan Collaborative Cohort Study for Evaluation of Cancer Risk (JACC) study supported by Ministry of Education, Science, Sports and Technology examined mainly risk for cancer and mortality, subjects of these two cohort were both over 100,000;^10^ and Sato et al. examined incidence of stroke and cardiovascular diseases with a cohort of about 12000 subjects in mainly urban area.^12^ Compared with these studies, the present study characterized as following; most of participant districts located in mainly rural areas; local governments and municipal hospitals and clinics are co-operated with this study and physicians of these join, so follow-up loss of incidence of stroke and cardiovascular diseases are very low; and after registration of incidence, independent diagnosis committee are organized.

Hatano et al.^22^ reported the SMR for strokes was negatively associated with meat, milk, eggs and bread consumption, and positively associated with salted and dried fish, alcohol beverages and cakes in 1969-1983. The present study was the first study to which has calculated the SMRs of population-based multi-communities prospective cohorts. The SMRs of the cohort subjects in the JMS Cohort Study were 0.68 for males and 0.73 for females. Jones et al.^23^ demonstrated the bias in the SMR using the general population. In that study, the authors found that bias may be a major problem, causing substantial underestimation of the true relative risk, when either the prevalence of exposure in the general population or the SMR are large. Participants in a health checkup examination, not randomized sampling, may have some biases. The most important bias is selection bias. If the response rate is low, the participants may be selected even if the selection was either intended or not. The response rate in the present study was 62.7%, which was higher than that of a previous study (about 30%). The SMR of the current study was higher than that of Shirasaki’s. This may be due to the higher response rate in this study,^13^ but selection bias exists to some extent, we should be careful to use these data.

In comparison with the fourth national survey of circulatory disorder, which was carried out in 1990 in 300 randomly selected districts of Japan,^24^ mean values of systolic and diastolic blood pressures, total cholesterol, and HDL-cholesterol were slightly lower in the present study than those in the survey for males (JMS cohort Study and the survey: systolic blood pressure; 131.4mmHg and 137.6mmHg, diastolic blood pressure; 79.2mmHg and 83.6mmHg, total cholesterol; 184.9mg/dl and 198.6mg/dl, HDL-cholesterol; 48.8mg/dl and 50.9mg/dl, respectively). For women, a similar tendency was observed. Methods of blood pressure measurement were different between the two studies, so comparability may be limited, but lipids were measured by the same method. The discrepancies in data between the two populations may be due to some selection bias and it seemed the present study subjects were slightly healthier. We reported general characteristics using cross-sectional analysis of the baseline data, and also examined the SMRs of the cohort subjects. Although some selection bias exists, the subjects of the JMS Cohort Study could be regarded to represent general population of Japan.

## APPENDIX

The Jichi Medical School (JMS) Cohort Study Group; Akizumi Tsutsumi (Department of Hygiene, Okayama University School of Medicine, Okayama), Atsushi Hashimoto (Aichi Prefectural Aichi Hospital, Aichi), Eiji Kajii (Department of Community and Family Medicine, Jichi Medical School, Tochigi), Hideki Miyamoto, Hidetaka Akiyoshi (Department of Pediatrics, Fukuoka University School of Medicine), Hiroshi Yanagawa (Saitama Prefectural University, Saitama), Hitoshi Matsuo (Gifu Prefectural Gifu Hospital, Gifu), Jun Hiraoka (Tako Central Hospital, Chiba), Kaname Tsutsumi (Kyushu International University, Fukuoka), Kazunori Kayaba (Joetsu Medical Center, Niigata), Kazuomi Kario (Department of Cardiology, Jichi Medical School, Tochigi), Kazuyuki Shimada (Department of Cardiology, Jichi Medical School, Tochigi), Kenichiro Sakai (Akaike Town Hospital, Fukuoka), Kishio Turuda (Takasu National Health Insurance Clinic, Gifu), Machi Sawada (Agawa Osaki National Health Insurance Clinic, Kochi), Makoto Furuse (Department of Radiology, Jichi Medical School, Tochigi), Manabu Yoshimura (Kuze Clinic, Gifu), Masahiko Hosoe (Gero Hot-Spring Hospital, Gifu), Masahiro Igarashi, Masafumi Mizooka (Kamagari National Health Insurance Clinic, Hiroshima), Naoki Nago (Tsukude Health Insurance Clinic, Aichi), Nobuya Kodama (Sakugi Clinic, Hiroshima), Noriko Hayashida (Tako Central Hospital, Chiba), Rika Yamaoka (Awaji-Hokudan Public Clinic, Hyogo), Seishi Yamada (Wara National Health Insurance Hospital, Gifu), Shinichi Muramatsu (Department Neurology, Jichi Medical School, Tochigi), Shinya Hayasaka (Department of Community and Family Medicine, Jichi Medical School, Tochigi), Shizukiyo Ishikawa (Department of Community and Family Medicine, Jichi Medical School, Tochigi), Shuzo Takuma (Akaike Town Hospital, Fukuoka), Tadao Gotoh (Wara National Health Insurance Hospital, Gifu), Takafumi Natsume (Oyama Municipal Hospital, Tochigi), Takashi Yamada (Kuze Clinic, Gifu), Takeshi Miyamoto, Tomohiro Deguchi (Akaike Town Hospital, Fukuoka), Tomohiro Saegusa (Sakuma National Health Insurance Hospital, Shizuoka), Yoshihiro Shibano (Saiseikai Iwaizumi Hospital, Iwate) Yoshihisa Ito (Department of Laboratory Medicine, Asahikawa Medical College, Hokkaido), and Yosikazu Nakamura (Department of Public Health, Jichi Medical School, Tochigi).
